# Loss of STAT3 in osteoblasts has detrimental and sexually dimorphic effects on skeletal development

**DOI:** 10.1371/journal.pone.0315078

**Published:** 2024-12-17

**Authors:** Rebecca K. Davidson, Kylie Corry, Amos Orlofsky, Ping Li, Caleb E. Russell, Amy Zhang, Mariana Moraes de Lima Perini, Carlie N. Priddy, Andrew V. Nguyen, Jiliang Li

**Affiliations:** 1 Department of Biology, Indiana University Indianapolis, Indianapolis, Indiana, United States of America; 2 Department of Biological Sciences and Geology, the City University of New York-Queensborough Community College, Bayside, New York, United States of America; 3 Department of Surgery, Indiana University School of Medicine, Indianapolis, Indiana, United States of America; University of Vermont, UNITED STATES OF AMERICA

## Abstract

Studies with genetically modified mice have implicated the transcriptional regulator STAT3 as a key modulator of bone development. STAT3-OKO knockout mouse lines were generated in two genetic backgrounds, pure C57BL/6 (STAT3-OKO-BL) and mixed C57BL/6, CD1 (STAT3-OKO-M). Both lines exhibited defective postnatal bone development resulting in reduced body weight and shortened femurs that displayed low bone mineral density as well as cortical widening and thinning in the diaphyseal region. Remarkably, each of these defects displayed sexual dimorphism that was dependent on genetic background: the phenotype was entirely male-specific in STAT3-OKO-M but not in STAT3-OKO-BL, in which defects were similar in both sexes. However, both lines exhibited a male-specific bone defect in mineralization, and also in bone mechanical properties related to bone quality, such as yield stress and ultimate stress. On the other hand, bone mechanical properties such as ultimate force, that may reflect density and macrostructure rather than bone quality, showed male-specific defects only in STAT3-OKO-M. These findings suggest that STAT3 may regulate multiple sex-dependent mechanisms in bone development that control either mineralization or bone accrual, and that the sex-dependence of at least some of these mechanisms is affected by genetic background. Finally, we used CRISPR/Cas9 to generate STAT3-deficient preosteoblastic cells from immortalized wild-type bone marrow stem cells and showed that the defective osteoblastic differentiation of STAT3-ablated cells was associated with reduced gene expression of Wnt3a and Wnt5a, consistent with other studies that identify Wnt signaling pathways as potential effector mechanisms for STAT3-mediated regulation of bone development.

## Introduction

The structural organization and mechanical properties of bones are dependent on a continual dynamic balance between the processes of bone formation by osteoblasts and resorption by osteoclasts, with osteocytes playing a coordinating role. While the signals that control the maturation and function of these cells are not fully understood, all three cell types are regulated by cytokines of the IL-6 family, which share the feature of downstream signaling through JAK/STAT mediators [1]. In particular, the transcriptional regulator STAT3 has been implicated in the maturation and/or function of each of the three cell types. Recently, multiple studies from our and other laboratories have demonstrated a critical role for STAT3 in promotion of normal bone development, by genetic deletion of STAT3 with Cre recombinase expression driven by a variety of promoters that have variable cell- and stage-specificity within the osteoblast, osteocyte, osteoprogenitor and mesenchymal lineages [2–6]. These studies strongly implicate STAT3 expression in the osteoblast/osteoprogenitor lineage in the support of normal bone growth.

A question left unresolved by these reports is the nature of the specific bone developmental processes that are dependent on osteoblast-lineage expression of STAT3. The skeletal defects that have been documented in STAT3-deleted mice might potentially stem from deficiencies in either bone mass accrual, reflected in extrinsic properties such as bone dimensions, geometry, porosity, density and strength, or alternatively in bone material properties, reflected in intrinsic properties such as matrix composition, as well as biomechanical functions that depend on those intrinsic properties, such as elasticity and ultimate stress.

In the current work, we utilize the osterix promoter to drive Cre expression in immature osteoprogenitor cells, the precursors of the osteoblast lineage, to remove STAT3 signaling from osteoblast development as completely as possible. We demonstrate for the first time that the structural alterations in bone formed by STAT3-deficient osteoblasts result in mechanical deficiencies. Furthermore, we describe a novel finding of strain-dependent sexual dimorphism with respect to both the structural and mechanical bone phenotypes resulting from STAT3-deletion. Finally, we demonstrated a potential role of osteoblast STAT3 in the promotion of signaling through the Wnt pathway, a key signal transducing system in bone formation.

## Materials and methods

### Experimental animals

All mouse studies were reviewed and approved by the Indiana University Purdue University Indianapolis, School of Science Institutional Animal Care and Use Committee (IACUC). Osteoblast-specific STAT3 knockout mice were generated by crossing mice with floxed Stat3 (STAT3^flox/flox^) in C57BL/6 background (a kind gift from Dr. Xin-Yuan Fu in the Department of Microbiology and Immunology, Indiana University School of Medicine) and Osterix-Cre (Osx-Cre) mice from Jackson Laboratory (Bar Harbor, ME). For the generation of the STAT3-OKO-M line, the Osx-Cre mice were in a mixed C57BL/6J, CD1 background. In the subsequent generation of the STAT3-OKO-BL line, the Osx-Cre had been backcrossed to create a pure C57BL/6J background. Cre^+/-^, STAT3^+/flox^ heterozygotes were crossed to generate experimental STAT3-OKO mice as well as Cre^+/-^, STAT3^+/+^ controls. Control mice in all experiments are Cre^+/-^, STAT3^+/+^, since skeletal phenotypes can result from the Osx:Cre transgene [7]. Animals were euthanized via CO_2_ inhalation followed by cervical dislocation. Femurs were stripped of soft tissue and the left femurs were wrapped in saline-soaked gauze and stored at -20°C for PIXImus, microCT and 3-point bending studies. The right femurs were immersed in 10% neutral buffered formalin overnight for histology and histomorphometry.

### Whole mount of newborn mice

Newborn mice at 36 hours postpartum were collected and euthanized. The sex of the pups was initially determined by visual assessment of scrotal pigmentation [8] and confirmed by PCR using the following primers to detect Smcx and Smcy genes: 5’- CCGCTGCCAAATTCTTTGG -3’; 5’- TGAAGCTTTTGGCTTTGAG -3’. These primers generate amplicons of distinct sizes from X-linked Smcx and Y-linked Smcy [9]. The pups were fixed in 95% ethanol for 1–2 days and fat was removed by acetone immersion prior to staining. The pups were stained with 0.005% alizarin red and 0.015% alcian blue 8GX in 5% glacial acetic acid/70% ethanol for 2–3 days. Afterward, they were washed with 95% ethanol for one hour and muscle tissue removed using 1% KOH. Lastly, the pups were passed through a series of glycerol/KOH washes and then stored in 80% glycerol. All pups were imaged using a Stereologer system (Stereology Resource Center, Chester, MD) on a Nikon SMZ1500 microscope.

### Bone mineral density measurement (PIXImus)

Bone mineral density (BMD, g/cm^2^) and bone mineral content (BMC, g) were determined for the left femurs of the experimental mice. BMD and BMC were measured and calculated by using peripheral dual-energy X-ray absorptiometry (pDXA, PIXImus II, GE-Lunar Co.).

### Micro-computed tomography (μCT) analysis

Left femurs were slowly brought to room temperature and then wrapped in parafilm. The femurs were positioned and stabilized in Styrofoam and mounted on the micro-CT platform for scanning (SkyScan 1172, Bruker-microCT, Kontich, Belgium). Scanning parameters were set as follows: voltage: 60 kV, resolution: 12 μm, binning mode: 2K, filter: Al 0.5 mm, rotation step: 0.7°, and averaging frame: 2. The femur specimens were rotationally scanned for 180° to generate raw projection images per rotation step (0.7°). Once scanned, the raw images were reconstructed using the SkyScan NRecon software (Bruker-microCT, Kontich, Belgium) with the following parameters: Post alignment: variable, Smoothing: 2, Ring artifact reduction: 5, Beam hardening: 20, Threshold: 0–0.11 and File format: BMP. Reconstructed images were analyzed using SkyScan CT-Analyser (CTAn) (Bruker-microCT, Kontich, Belgium) software. Three dimensional representations of the midshaft cortical and distal trabecular regions were generated by first selecting the region of interest in the SkyScan CT-Analyser software and then exporting as “3D model” in the binary images view. The file was then opened in the Skyscan CT-vol program for 3D manipulation and imaging.

### Mechanical testing

After μCT scanning was complete, the left femurs were subjected to 3-point bending as previously described [10]. The femurs were immersed in phosphate buffered saline (pH 7.4) on petri dishes during the test to prevent drying. The femurs were loaded anterior side upward onto a Bose ElectroForce 3200 Test Instrument, pre-loaded to a force of 0.2–0.5 N, pre-conditioned for 8 cycles (2 Hz), and then monotonically tested to failure at a rate of 0.025 mm/s up to 30 N or failure. During each bending test, the load and deformation were recorded every 0.04 seconds. From these recordings, structural strength (yield force and ultimate force), stiffness (the slope of the linear portion of the force vs. displacement curve), and deformation (yield deformation, failure deformation and post-yield deformation) were derived at the whole bone level [11, 12]. The point of fracture initiation was noted and measured on the anterior face from the proximal end of the femur.

To derive structural-level mechanical properties, the prior micro-CT data from the same femurs was used to normalize the geometry of the bones. Seven slices from the 3D reconstruction of the left femur, including the failure site and three slices proximally and distally, were analyzed using a MATLAB script [13, 14]. The derived stress-strain data were used to calculate the following bone material properties: ultimate stress, ultimate strain and elastic modulus.

### Histology and histomorphometry

Eight week old mice were injected i.p. with calcein (30 mg/kg body weight, Sigma-Aldrich) seven days prior to sacrifice, and five days later with alizarin (50 mg/kg body weight, Sigma-Aldrich). Distal portions of formalin-fixed right femurs were embedded in methyl methacrylate and processed. A total of three 4 μm sections from each mouse were obtained: two unstained slides for dynamic histomorphometry and one von Kossa slide for osteoid/osteoblast scoring.

For dynamic histomorphometry, thin sections of trabecular bone at the distal femur were left plasticized and coverslipped using Eukitt mounting reagent. The fluorescent labels were visualized using an Olympus BX53 light/fluorescent microscope and Olympus DP72 camera interfaced with Osteomeasure^™^ software version 1.01 (OsteoMetrics Inc, Decatur GA). An area of approximately 0.6 mm^2^ was measured, located 0.4 mm proximal to the growth plate and 0.5 mm medial to the endosteal surface of the cortical bone. All measurements were taken at 200x magnification under fluorescent light. Abbreviations are according to Parfitt et al. [15].

For static histomorphometry, slides were deplasticized in acetone and measurements performed at 200x magnification in an area 0.4 mm proximal to the distal growth plate. TRAP-stained slides were counterstained with toluidine blue [16]. The following primary data were collected through the OsteoMeasure system: bone area (B.Ar), total perimeter (B.Pm), osteoclast number (Oc.N), osteoclast surface (Oc.S), osteoid thickness and osteoid surface (OS). These primary data were used to derive osteoid volume (OV), total volume (TV) and their ratio (OV/TV).

### Generation of immortalized STAT3 knockout cell line

**Fig 1:**
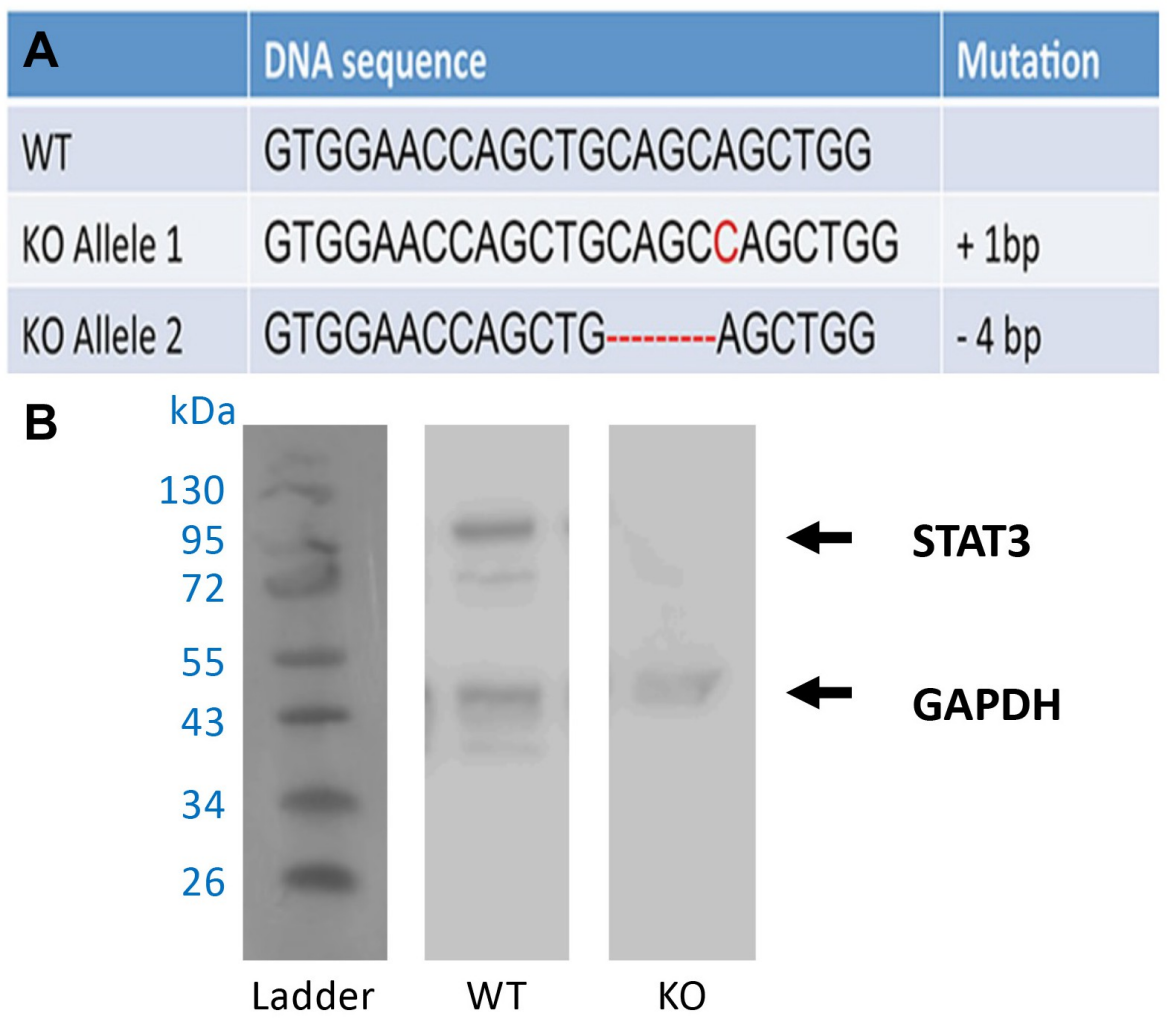
Establishment of STAT3-ablated (STAT3-KO) immortalized bone marrow stromal cells (BMSC). (**A**) Indel mutations revealed by DNA sequencing of STAT3 alleles in STAT3-KO cells generated with the CRISPR/Cas9 system. Mutations are indicated in red. (**B**) Western blot analysis confirmed the loss of *Stat3* gene expression. (Lane 1: protein ladder; Lane 2: wild type control BMSC; Lane 2: STAT3-KO BMSC).

Bone marrow stromal cells (BMSC) were harvested from 6-8-week old control mice (Cre^+/-^, STAT3^+/+^) and were immortalized utilizing murine telomerase reverse transcriptase (mTERT) plasmid packaged into viral particles (Bidwell Laboratory, Indiana University School of Medicine, Indianapolis, Indiana) [17]. Briefly, the BMSCs were seeded at 6,000 cells/mm^2^ and maintained in α–MEM culture medium for 10–14 days before being exposed to the viral supernatant. Surviving cells were selected, isolated, and expanded. Once the wild-type BMSCs were immortalized, they were additionally modified using the CRISPR/Cas9 gene editing tool to generate a deletion in the Stat3 gene [18]. The BMSC cells were transfected with a linearized plasmid generated from the Cas9-expressing vector pX330/Bbs1(Addgene) [19] by insertion of a single-guide RNA targeting the second exon of Stat3. The cells were then screened using PCR and DNA sequencing to identify and isolate STAT3 knockout (KO) cell lines and confirmed with Western blot (Fig 1).

### Cell proliferation assay

Immortalized wild-type and STAT3-OKO BMSCs were seeded at an initial density of 100,000 cells/well in a 6-well culture dish. Cells were cultured for 72 hours in α-MEM medium with 10% fetal bovine serum. Cells were then counted after the 72 hours using a hemacytometer.

### Western blot

Protein samples (about 10 ug) were separated by gel electrophoresis and transferred to a nitrocellulose membrane, which was then probed with anti-STAT3 (Cell Signaling Technologies, Danvers, MA) and anti-GAPDH antibody (Sigma), as previously described [20]. The original images of Western blot are included in S1 Raw images.

### RT-qPCR

RNA was isolated using TRIzol Reagent Kit (Thermo Fisher Scientific, Waltham, MA) and purified using RNeasy Mini Kit (Qiagen, Hilden, Germany) according to the manufacturer’s protocol. Total RNA concentration was determined using NanoDrop (Thermo Fisher Scientific, Waltham, MA). cDNA was synthesized from total RNA using Superscript III mix (Thermo Fisher Scientific, Waltham, MA). Gene expression was analyzed by real time qPCR using SYBR Green PCR Master Mix (Applied Biosystems, Forster City, CA) and primers specific to both Wnt3a and Wnt5a genes: Wnt3a Forward: CTCCTCTGCAGCCTGAAGC, Wnt3a Reverse: GTGGACGGTGGTGCAGTT; Wnt5a Forward: GTGGTCGCTAGGTATGAATAA, Wnt5a Reverse: CGCGTATGTGAAGGCCGTC).

### Alkaline phosphatase staining

A modified version of the Sigma-Aldrich Alkaline Phosphatase Staining protocol was utilized to stain immortalized BMSCs. Briefly, BMSCs were cultured in osteogenic media containing 50 μg/ml ascorbic acid. Upon nearing confluency, the cells were fixed using 4% phosphate-buffered formalin and then incubated in an alkaline dye mixture containing Fast Blue RR Salt and Napthol AS-MX Phosphate Alkaline solution for 30 minutes in the dark. Lastly, the cells were incubated in Mayer’s Hematoxylin solution to counterstain and evaluated microscopically for regions of alkaline phosphatase staining.

### Statistical analysis

All data are reported as mean ± SD (standard deviation). Outliers were removed using Dixon’s test. The differences between groups were assessed by t-tests that in some cases were Bonferroni-corrected for the presence of two independent variables, in which case the threshold p value for significance (alpha) was reduced from 0.05 to 0.025. T tests were two-tailed, except for BMD. For BMD one-tailed tests were considered appropriate, since prior observation of skeletal defects upon STAT3 depletion, both in this study and in earlier work, made the likelihood of BMD increase negligible in knockout mice. For measurements carried out in separate experiments on backcrossed and non-backcrossed mice, a combined p value was obtained by Fisher’s method, using the Metap package in R [21]

## Results

### Loss of STAT3 in osteoblasts impairs postnatal bone growth in a sex- and strain-dependent manner

We initially examined the effects of osteoblast STAT3 depletion in mice of mixed BL6;CD1 background. Subsequently, backcrossing of Osx:Cre mice to BL6 afforded the opportunity to repeat many of the studies in a pure BL6 background. We refer to these knockout strains respectively as STAT3-OKO-M (mixed BL6;CD1) and STAT3-OKO-BL (BL6 background). Control mice are in all cases Cre+, due to the potential for skeletal phenotypes resulting from the Osx:Cre allele [7].

**Fig 2:**
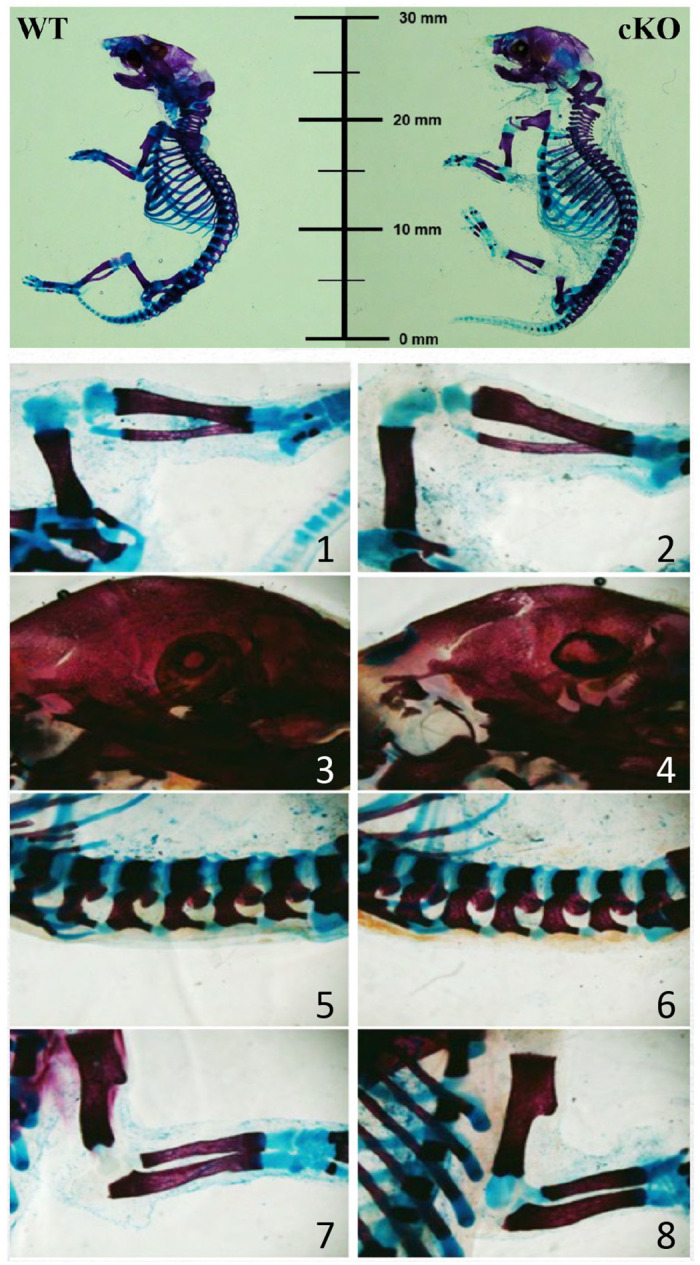
Whole skeletal preparation of newborns. Alcian blue was used to stain sulphated glycosaminoglycans in cartilage and alizarin red for hydroxyapatite in bones. Representative male skeletons are displayed for wild type (WT) control and STAT3-OKO-M pups (Top two panesl). Zoomed views of the control (1, 3, 5 and 7) and STAT3-OKO-M (2, 4, 6, and 8) skeletons, showing patellar and tarsal joints (1 and 2) calvaria (3, 4), lumbar vertebrae (5 and 6), and elbow and carpal joints (7 and 8).

We did not observe a significant difference between newborn STAT3-OKO-M mice and controls with respect to bone size except a slight delay in mineralization of osteoid in STAT3-OKO-M mice in comparison with the controls (Fig 2). We then proceeded to examine adult mice. In previous studies of skeletal effects of STAT3 knockout via Osx-Cre, effects on female mice were not reported [7], although when three other promoters were used to delete STAT3, similar skeletal phenotypes were observed in male and female mice [7]. We therefore hypothesized that STAT3 knockout via Osx-Cre would similarly display a sex-independent skeletal phenotype. Since this hypothesis requires a joint rejection of the individual null hypotheses for separate male and female analyses, alpha adjustment for multiple comparisons is not performed [22].

**Fig 3:**
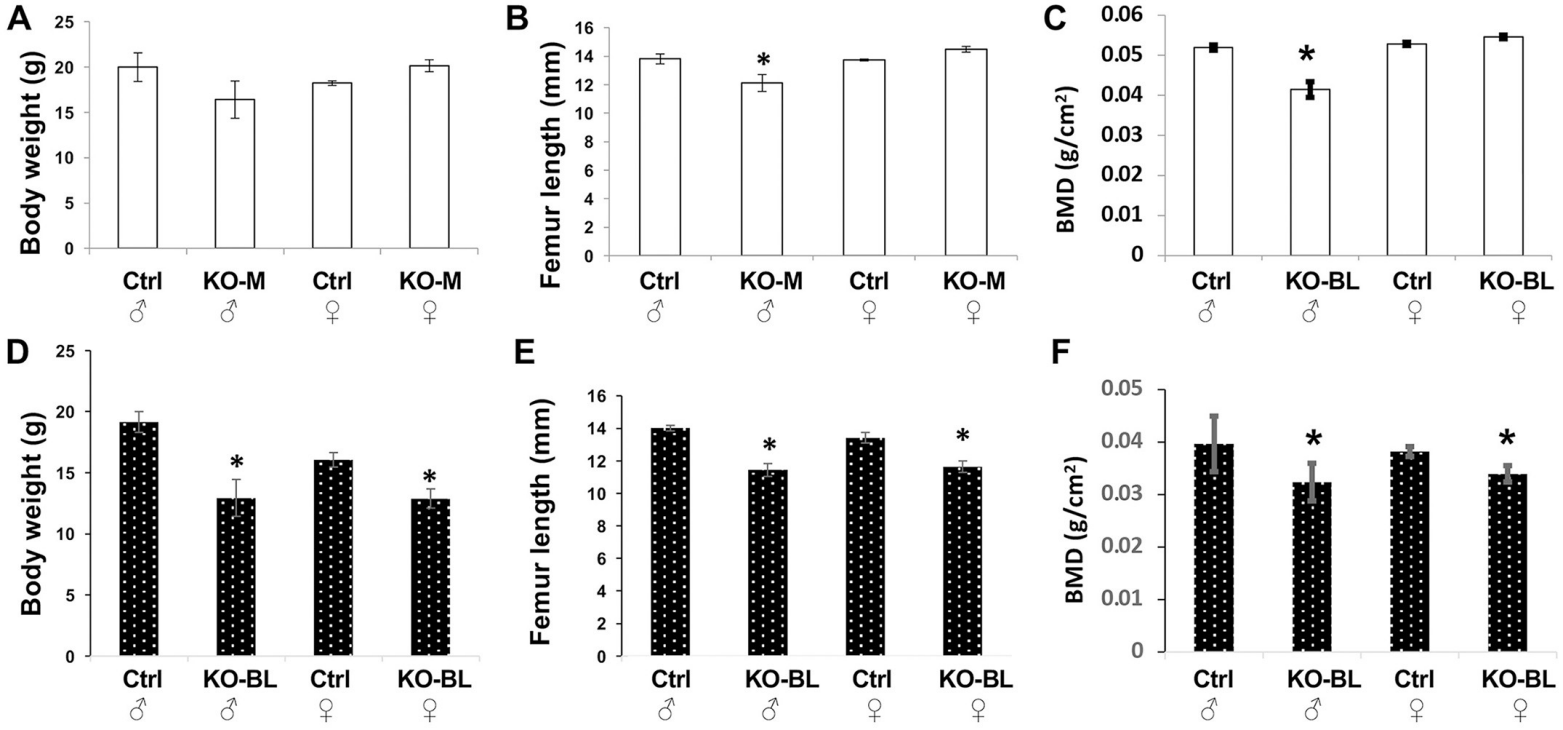
Gross characteristics of STAT3-KO mice. Body weight (**A** and **D**), femur length (**B**, and **E**) and bone mineral density (BMD) (C and F) were assessed in 8-week old mice of the indicated sex (♂ = male and ♀ = female). Controls (A-C) were compared to either STAT3-OKO-M mice (KO-M; mixed background) or STAT3-OKO-BL mice (KO-BL; C57BL/6 background) (D-F). Total n = 27 mice (range 4–9). * p < 0.05.

As expected from previous studies, gross defects in body weight and femur length were observable in male STAT3-OKO-BL mice at eight weeks of age (Fig 3). In agreement with hypothesis, similar effects were also observed in female mice. In STAT3-OKO-M mice, the expected male defects were confirmed, although with respect to body weight the effect did not achieve statistical significance (Fig 3). However, no effects were observed in female mice with respect to either body weight or femur length, suggesting the possibility of sexual dimorphism for these effects in the mixed background.

Skeletal defects in STAT3 knockout mice have been associated with an osteoporotic phenotype characterized by reduced bone mineral density (BMD) [7]. BMD was examined in control and STAT3-OKO femurs using dual-energy X-ray absorptiometry. As expected from the prior study, we observed BMD reduction in male STAT3-OKO-M and STAT3-OKO-BL mice, but once again a female effect was observed only in STAT3-OKO-BL and not in STAT3-OKO-M (Fig 3C and 3F).

**Fig 4:**
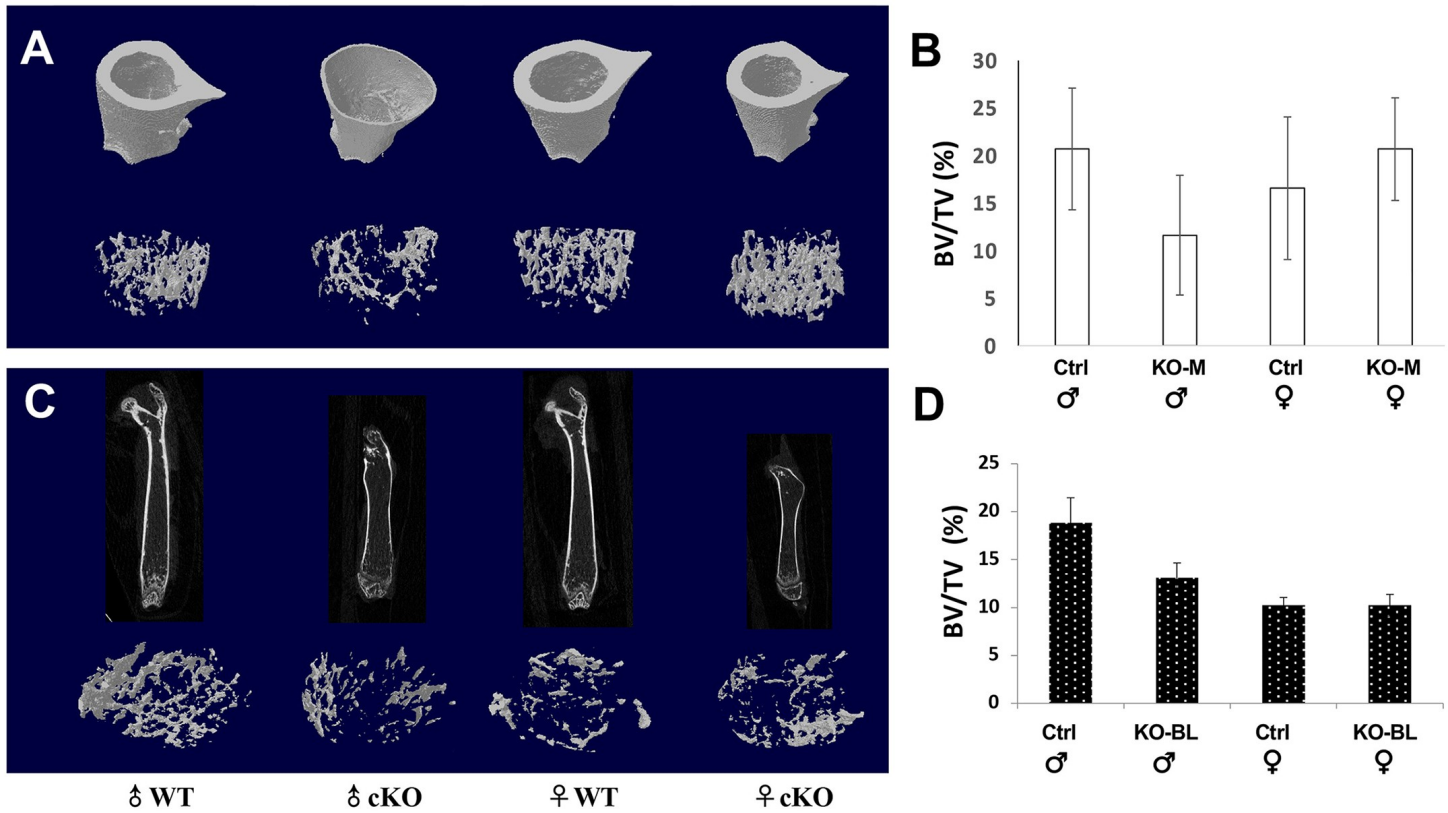
Femoral structures (cortical bone and distal trabecular bone) in STAT3-OKO mice. (**A**) femoral structure of STAT3-OKO-M (KO-M) and (**C**) femoral structure of STAT3-OKO-BL mice of the indicated sex and compared to their respective controls. There was no significant difference in distal trabecular bone volume (BV/TV) between wild type controls and STAT3-OKO mice. Total n (KO-M + Ctrl) = 16 (range 2–5). Total n (KO-BL + Ctrl) = 27 (range 4–9).

Notably, all STAT3-OKO-BL femurs and all male STAT3-OKO-M femurs displayed increased width of the femoral shaft in a region distal to the proximal growth plate (Fig 4A and 4C). further analysis of μCT images demonstrated that trabecular bone volume (BV/TV) at the distal femurs was slightly lower in both male STAT3-OKO-M and STAT3-OKO-BL mice than the male control mice (Fig 4B and 4D). The BV/TV between female STAT3-OKO-M and STAT3-OKO-BL mice was similar to the female control mice. These data suggest that STAT3 deficiency in osteoprogenitors affect cortical development more than trabecular bone, especially in male mice.

### STAT3-OKO mice display a sex-dependent defect in osteoid mineralization

**Fig 5:**
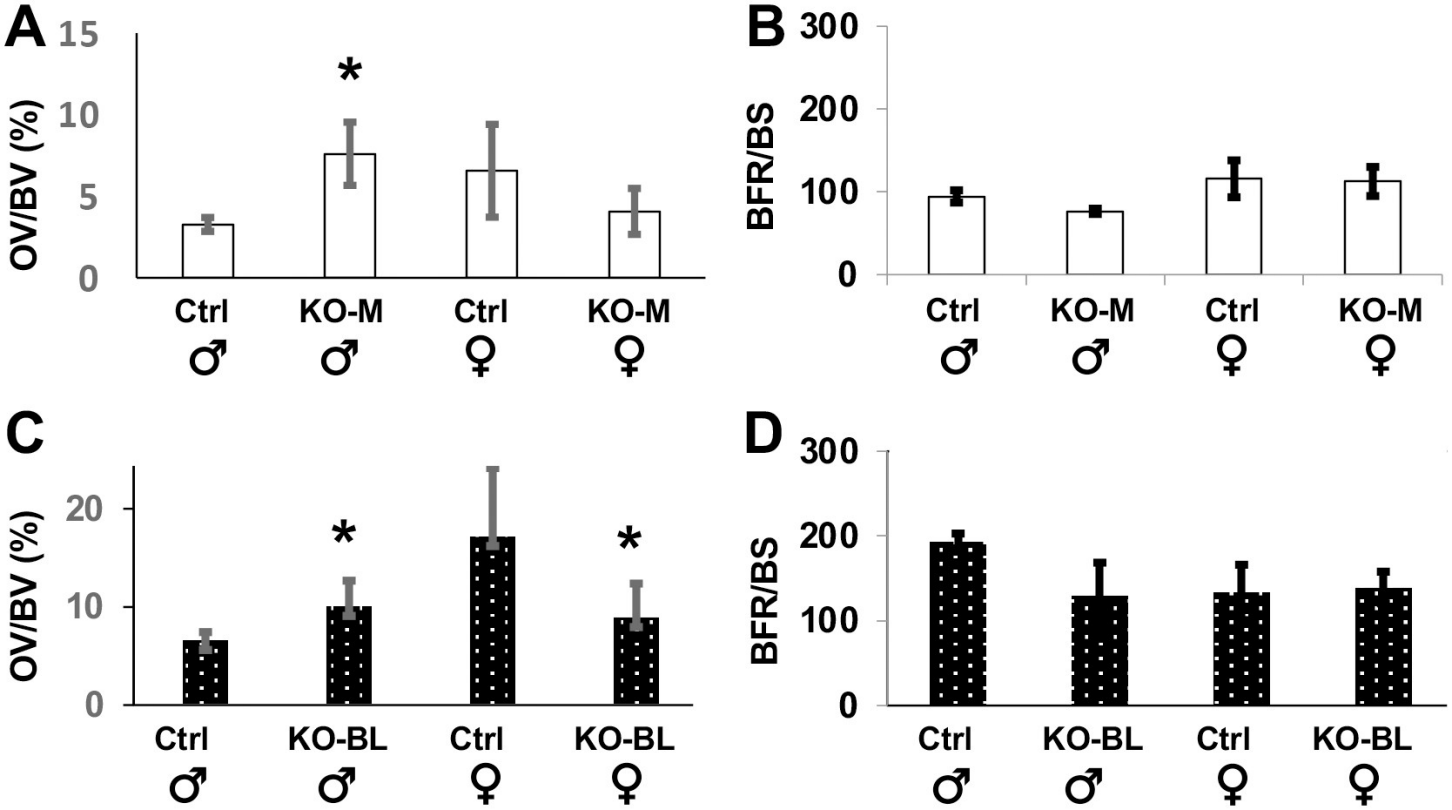
Osteoid volume and bone formation rate in STAT3-OKO femurs. Von Kossa staining was used to assess osteoid volume/bone volume (OV/BV, A and C) and calcein/alizarin fluorescent labels were used to assess bone formation rate/bone surface (BFR/BS, B and D) in trabeculae of STAT3-KO-M (A and B) and STAT3-OKO-BL (C and D) mice. Total n (KO-M + Ctrl) = 17–18 (range 3–5). Total n (KO-BL + Ctrl) = 27 (range 4–9). * p < 0.05.

To further analyze the bone composition of femurs of the knock-out mice, we assessed mineralization by measurement of osteoid volume (OV/BV) and osteoid surface (OS/BS) in trabecular bone in both STAT3-OKO-M and STAT3-OKO-BL mice compared to controls. Inspection of the results revealed a consistent pattern of sexual dimorphism, such that for every measurement the effect of STAT3 deletion was in opposite direction for male and female mice (Fig 5A and 5C). We therefore were able to examine the hypothesis of male-specific effect by limiting statistical testing to male mice, without adjustment of alpha, since the null hypothesis of non-dimorphic effect does not need to be tested. Significant male-specific increase in osteoid was detected in all cases, except that the increase in OV/BV for STAT3-OKO-BL did not achieve significance (p = 0.08). The aggregate significance of the effect of STAT3 deletion in the two mouse populations (males only) was determined using Fisher’s combined probability, yielding p = 0.010 for OV/BV data and p = 0.026 for OS/BS data.

An increase in OV and OS could potentially reflect a defect in mineralization or alternatively an increased rate of bone formation. We therefore examined trabecular bone formation rate (BFR/BS) in the distal femur by dynamic histomorphometry and observed no effect of STAT3 ablation in either STAT3-OKO-M or STAT3-OKO-BL mice (Fig 5B and 5D). Taken together these findings suggest a delay in bone mineralization in STAT3-OKO mice.

### STAT3-OKO does not present a significant increase in bone resorption

**Fig 6:**
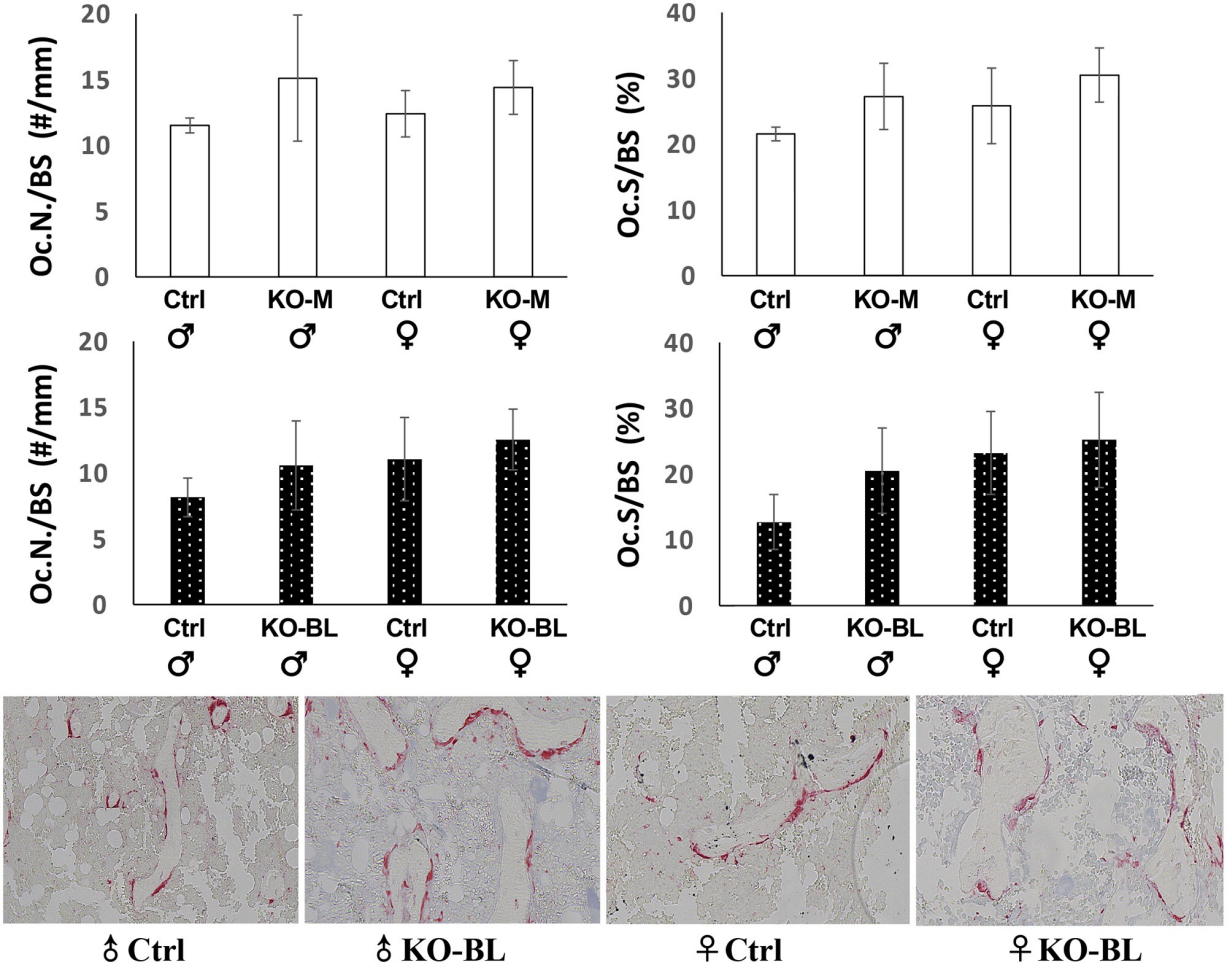
Osteoclast number and osteoclast surface assessed in both STAT3-OKO-M and STAT3-OKO-BL mice. Compared to the wild type controls, both osteoclast number per bone surface (Oc.N/BS) and osteoclast surface per bone surface (Oc.S/BS) did not increase significantly in either male and female STAT3-OKO-M or STAT3-OKO-BL mice. TRAP stain was used to stain osteoclasts in trabecular area of the distal femurs in male and female wild type controls and STAT3-OKO-BL mice. Total n (KO-M + Ctrl) = 17 (range 3–5). Total n (KO-BL + Ctrl) = 24 (range 3–8).

Both STAT3-OKO-M and STAT3-OKO-BL mice were examined with respect to bone resorption in trabecular bone at the distal femurs. Compared to the wild type controls, both osteoclast number per bone surface (Oc.N/BS) and osteoclast surface per bone surface (Oc.S/BS) did not increase significantly in either male and female STAT3-OKO-BL mice (Fig 6), suggesting that STAT3 deficiency in osteoprogenitors does not affect bone resorption during skeletal development.

### STAT3-OKO femurs display sex-dependent defects in extrinsic and intrinsic mechanical properties

**Fig 7:**
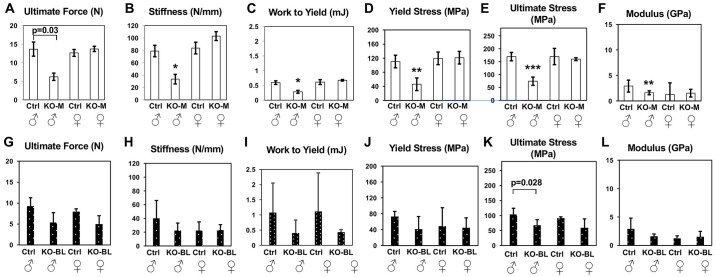
Biomechanical features assessed by three-point bending of control and STAT3-OKO femurs. (**A**-**F**) STAT-3-OKO-M (KO-M; mixed background) compared to the controls (Ctrl). Total n = 19 mice (range 3–6). (**G**-**L**) STAT3-OKO-BL (KO-BL; C57BL/6 background) compared to controls (Ctrl). Total n = 20 (range 4–6). * p < 0.025, **p < 0.005, ***p < 0.001. Bonferroni-corrected alpha is set at 0.025.

STAT3-OKO-M and STAT3-OKO-BL mice were both examined with respect to mechanical properties, which were not evaluated in prior studies of skeletal defects in STAT3 knockouts. Since the preceding results suggest potential defects in bone quality due to impaired mineralization, we examined extrinsic as well as intrinsic biomechanical parameters. Left femurs from 8-week control and STAT3-OKO mice were subjected to three-point bending to determine extrinsic properties (ultimate force, stiffness, work to yield) and three intrinsic properties (yield stress, ultimate stress, and elastic modulus). We again hypothesized that sex-independent defects would be observed, but no effect of STAT3 deletion was detected in females of either STAT3-OKO-M (Fig 7A–7F) mice or STAT3-OKO-BL mice (Fig 7G–7L).

Since the mechanical studies in STAT3-OKO-M mice all yielded highly significant effects (p<0.005), while the studies in STAT3-OKO-BL, despite similar effect sizes, did not achieve the 0.025 threshold, we sought to further confirm our prediction of defects in intrinsic parameters consistent with altered bone quality by determining whether meta-analysis of the combined studies yielded significant effects. Fisher’s combined probability was calculated for each of the three intrinsic parameters, and results were highly significant for each: yield stress (p = 0.0010), ultimate stress (p = 0.00026) and elastic modulus (p = 0.0039).

### STAT3 supports Wnt ligand expression and osteoblastic differentiation in preostoblastic cells

**Fig 8:**
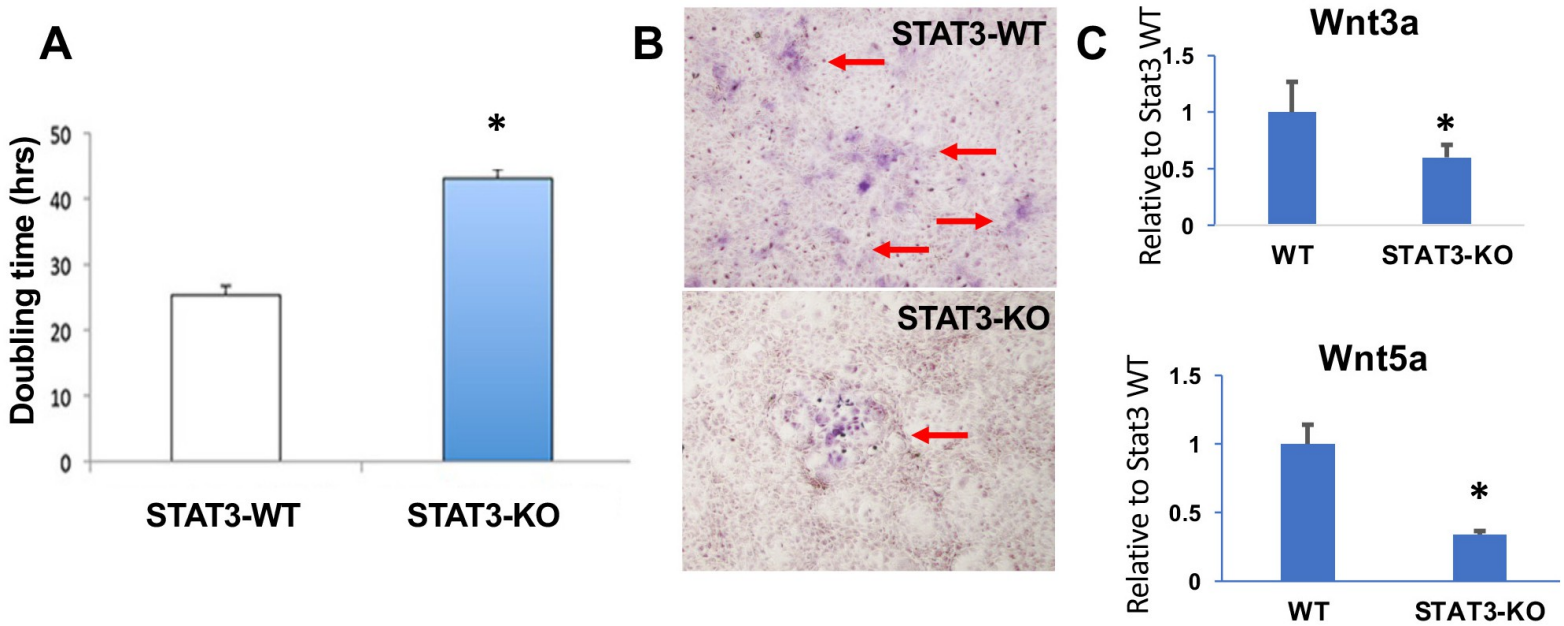
Proliferation, differentiation and Wnt ligand expression in STAT3-ablated (STAT3-KO) immortalized bone marrow stromal cells (BMSC). (**A**) Doubling time of control and STAT3-KO BMSC. * p < 0.01. (**B**) Alkaline phosphatase staining of control and STAT3-KO BMSC. (**C**) RT-qPCR analysis of Wnt ligand expression in control BMSC (WT) and STAT3-KO BMSC. * p < 0.05.

To further examine the role of STAT3 in the osteoblastic lineage, preosteoblastic bone marrow-derived stem cells (BMSC) were immortalized with mTERT and then edited with CRISPR/Cas9 in order to ablate STAT3 expression. A clonal cell line was isolated and identified as homozygous knockout for STAT3 by DNA sequencing, which demonstrated indel mutations in both alleles (Fig 1A). Immunoblot analysis confirmed the ablation of STAT3 expression (Fig 1B). Assessment of cell proliferation revealed a nearly two-fold decrease in proliferative rate in STAT3-KO cells (Fig 8A). When cells were placed in osteogenic culture medium to induce osteoblastic differentiation, STAT3-KO cells exhibited reduced differentiation as determined by alkaline phosphatase expression (Fig 8B).

Since the Wnt pathway is a potential downstream target of STAT3 [4], and Wnt signaling is a key regulator of bone development [4] we examined the dependence of Wnt ligand expression on STAT3 signaling in our immortalized BMSC lines. Gene expression of both Wnt3a and Wnt5a, which govern canonical and non-canonical Wnt pathway signaling, respectively, was determined in wild-type and STAT3-KO cells. As shown in Fig 8C, gene expression for each Wnt ligand was reduced in the absence of STAT3, consistent with a potential role for STAT3 in both canonical and non-canonical Wnt pathways that support osteoblastic function and bone homeostasis.

## Discussion

Assessment of fracture risk in osteoporosis patients has relied heavily on measurements of bone mass, specifically on BMD. However it is increasingly recognized that the predictive value of BMD is limited and may potentially be improved by consideration of other parameters relevant to bone quality, including intrinsic properties of bone material [23]. An important parameter of this kind is extent of mineralization, which has been shown to be critical in load-bearing performance [24]. The mineral content of bone shows a strong association with intrinsic mechanical properties, including the six properties we employed in this study: ultimate force, stiffness, work to yield, yield stress, ultimate stress and elastic modulus [25, 26]. Using sound transmission velocity as a surrogate for elastic modulus, it was shown that middle-aged women suffer from a loss of elasticity when compared with BMD-matched younger women [27]. A linkage between mineralization and intrinsic mechanical properties may therefore be relevant for an improved understanding of osteoporosis and other bone diseases. Our findings support this linkage, since we observe a close parallel between defects in the six mechanical parameters and excess production of osteoid relative to bone formation rate. More specifically, our study indicates that the role of STAT3, whose expression in the osteoblast lineage has been shown to be critical for the development of normal bone mass and geometry, should also be considered as a potentially critical factor for generation of the osteoblast-dependent bone material properties that are necessary for healthy bone structural behavior. Multiple studies have linked expression of STAT3 in cultured osteoblast lineage cells to mineralization (reviewed in [28]). Our findings are also consistent with earlier observations in which STAT3 deletion led to a reduction in bone formation rate but not to any change in osteoid volume [3].

Promoters used to drive Cre expression in the osteoblast lineage, including the Osx-Cre promoter used in this study, can also drive expression in osteocytes. We therefore need to consider the possibility that our findings reflect STAT3 functions in osteocytes. In an earlier study, we specifically targeted STAT3 deletion to osteocytes. The phenotype we observed was quite different from that in the current study, although there was a partial similarity with respect to bone mechanical properties. Osteocyte STAT3 knockout did not produce any gross skeletal deficiency, nor any alteration in BMD. Similar to this study, ultimate stress and elastic modulus were reduced by STAT3 depletion, but this reduction was similar in male and female knockout mice, in contrast to the dimorphism we observed. Furthermore, this reduction was only studied in fully grown mice at 18 weeks of age, in order to focus on the potential role of osteocytes in bone remodeling and adaptation to load, and it was accompanied by a dramatic decline in bone formation rate, which was not observed in the current study. Finally, the osteocyte STAT3 knockout mice showed reduction, rather than increase in osteoid accumulation, and no sexual dimorphism with respect to osteoid was observed. The alteration in intrinsic mechanical parameters observed in osteocyte STAT3 knockouts may reflect defective bone formation in the mature adult, as opposed to defects in bone development in the younger mice we examine here. However we cannot rule out that deletion of osteocyte STAT3 makes a contribution to the mechanical components of the phenotype we observe in the current study. We similarly cannot rule out a contribution from other lineages that can express Osx:Cre, including hypertrophic chondrocytes and others [29].

Our findings include a novel observation of sexual dimorphism with respect to the effects of osteoblast STAT3 depletion on bone development. In STAT3-OKO-M mice, both extrinsic and intrinsic mechanical properties were affected in a male-specific manner. In our earlier study of STAT3 deletion in osteoclasts, we demonstrated STAT3-dependent expression of multiple estrogen receptor-regulated genes in an osteoclastic cell line, potentially providing an explanation for our observation of female-specific defects in mice with osteoclast-specific deletion of STAT3 [20]. Studies in other systems have also observed sexually dimorphic phenotypes that may reflect interaction between STAT3 and sex hormone signaling. White et al for example, observed male-specific protection from medulloblastoma in STAT3 knockout mice, potentially related to male-specific enhanced expression of IL-6, which can activate STAT3 [30]. It has also been suggested that male-specific susceptibility to hepatocellular carcinoma is due to female protection via estrogen-mediated inhibition of IL-6 expression, as well as direct inhibition of STAT3 via estrogen-mediated expression of the tyrosine phosphatase PTPRO [31]. These studies lend support to the notion that our observation of male-specific STAT3-dependent development of bone mechanical properties may reflect estrogen-mediated antagonism of a subset of STAT3 signaling pathways.

Among the many potential effector pathways downstream of STAT3, the Wnt pathways are among the best characterized. In particular, multiple components of the canonical Wnt pathway, including Wnt coreceptors LRP5 and LRP6, as well as the downstream effector beta-catenin, have been shown to be critical for bone accrual in mice by osteoblast lineage-specific gene knockout [32]. The recent study of Yadav et al. showed that the perinatal skeletal defects in Osx-Cre STAT3-KO mice could be rescued by upregulation of beta-catenin signaling via inhibition of GSK3-beta. Non-canonical Wnt signaling, mediated by Wnt5a, may also play a role [33]. Because Wnt secretion by osteoblasts can potentially inhibit osteoclastogenesis [34], it may be that the osteoblast STAT3 function responsible for the phenotype in our strains is not the mediation of responses to Wnt signals in osteoblasts, but rather the stimulation of osteoblast Wnt production. This notion is consistent with our observation that STAT3 deficiency in an osteoblastic cell line leads to reduced gene expression of both Wnt3a and Wnt5a. In support of the idea that defective osteoblast Wnt secretion may be responsible for the STAT3-OKO phenotype, a severe defect in bone formation rate and bone accrual was observed in mice with an osteoblast-specific deletion of Wntless, which functions as a chaperone that is specifically essential for the secretion of Wnt ligands [35]. Notably, the defect did not appear until post-weaning stages, corresponding to our observation of a specifically postnatal phenotype.

The mounting connections between STAT3 signaling and osteogenic regulation are generating increasing avenues of investigation with a goal of developing treatments for osteoporosis, as well as other diseases of bone fragility. Our findings strengthen these connections by providing evidence linking osteoblast STAT3 function to mineralization and mechanical function. The signaling pathways that link STAT3 to these downstream functions remain to be investigated. Our profiling experiments have shown two key pathways involved the Wnt and Keap1-Nrf2 (data not shown) pathways. Overall, the findings reported here may assist in guiding future mechanistic studies to elucidate the intracellular signaling of osteoblasts and osteocytes involved in bone formation.

## Supporting information

S1 Raw images (PDF)
