# Supplementary material for: Loss of STAT3 in osteoblasts has detrimental and sexually dimorphic effects on skeletal development
Source: PLoS One. 2024 Dec 17;19(12):e0315078. doi: 10.1371/journal.pone.0315078 (PMC11651548; doi:10.1371/journal.pone.0315078)
Supplement: S1 Raw images — (PDF) [file pone.0315078.s001.pdf]

Immortalized STAT3 KO BMSC Lines confirmed by Western Blot

STAT3 KO lines: #14, #26 & #142

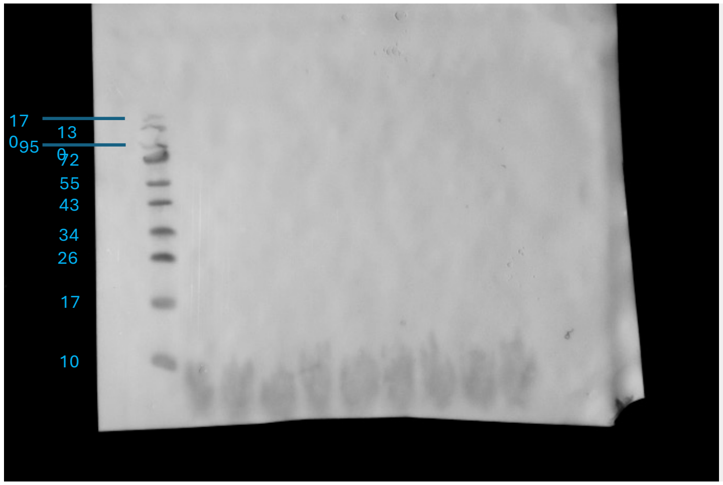

Figure 1: Protein Ladder (08/28/2013)

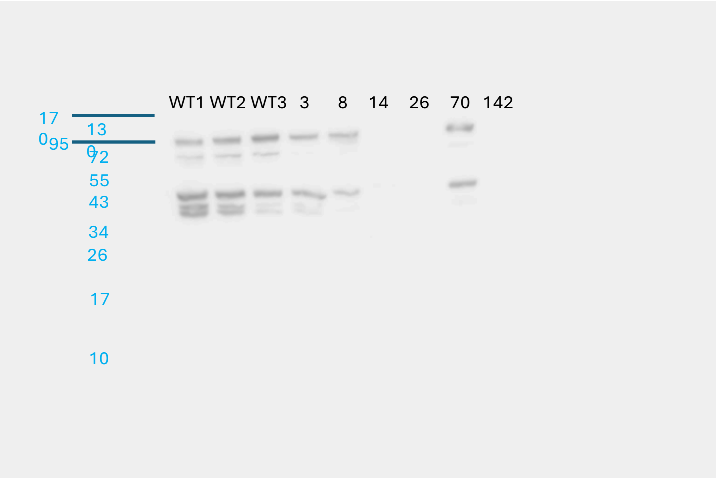

Figure 2: STAT3 (08/28/2013)

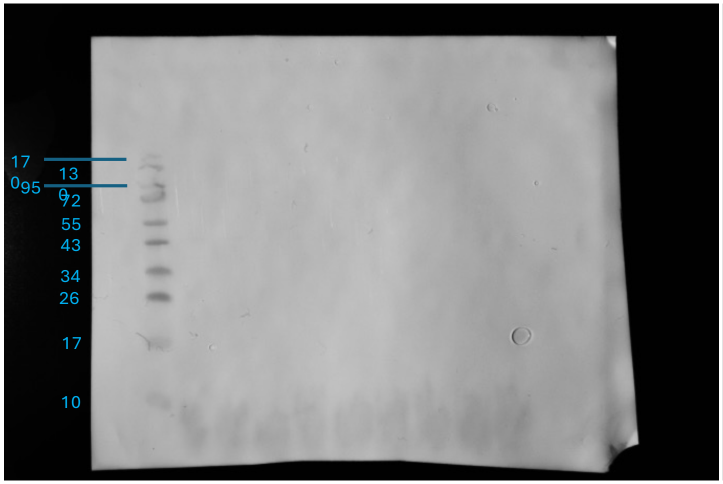

Figure 3: Protein Ladder (08/29/2013)

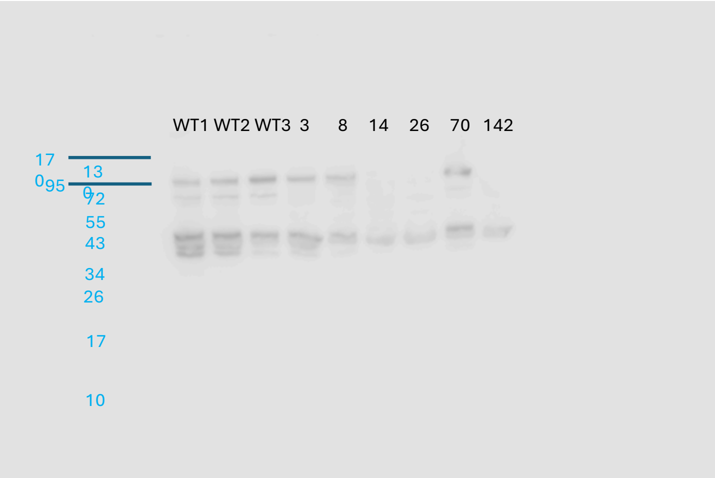

Figure 4: STAT3, GAPDH (08/29/2013)
